# Prevalence of *Coxiella burnetii *antibodies in Danish dairy herds

**DOI:** 10.1186/1751-0147-52-5

**Published:** 2010-01-21

**Authors:** Jens F Agger, Anna-Bodil Christoffersen, Erik Rattenborg, Jørgen Nielsen, Jørgen S Agerholm

**Affiliations:** 1 Department of Large Animal Sciences, Faculty of Life Sciences, University of Copenhagen, Groennegaardsvej 8, Frederiksberg, DK-1870 C, Denmark; 2 National Veterinary Institute, Technical University of Denmark, Bülowsvej 27, Copenhagen, DK-1790 V, Denmark; 3 Danish Cattle Federation, Udkjaersvej 15, Aarhus, DK-8200 N, Denmark

## Abstract

During recent years in Denmark higher rates of antibodies to *Coxiella burnetii *have been detected in animals and humans than previously reported. A study based on bulk tank milk samples from 100 randomly selected dairy herds was performed to estimate the prevalence and geographical distribution of antibody positive dairy herds. Using the CHEKIT Q-Fever Antibody ELISA Test Kit (IDEXX), the study demonstrated a prevalence of 59% antibody positive herds, 11% antibody intermediate herds and 30% antibody negative herds based on the instructions provided by the manufacturer. The geographical distribution does not indicate a relationship between the regional density of dairy farms and the prevalence of antibody positive dairy farms. The result supports the hypothesis of an increase in the prevalence of positive dairy herds compared to previous years.

## Findings

The bacterium *Coxiella burnetii *is a zoonotic agent and infection may cause Q fever in man and in animal species. The bacterium has been detected in a large number of animal species and with cattle, sheep and goats as being the most common reservoirs [1]. *C. burnetii *antibodies have been detected within cattle, sheep and goat herds in many countries of the world. Thus, selected publications that are valued as giving reliable estimates of the respective target populations reported a prevalence of antibody positive herds at 67% of Ontario dairy herds [2], 28% of Ontario sheep flocks [3], 21% of dairy herds in England and Wales [4], and more than 50% of Dutch dairy herds [5]. The prevalence of PCR positive herds was 40% of northern Italian dairy herds [6], and 22% of Basque dairy sheep herds [7].

Until recently, *C. burnetii *was considered to occur with a low prevalence in Denmark. However, testing for antibodies in cattle since 2003 indicated that the infection was widespread in cattle [8], and a recent study [9] found that 57% of 742 non-randomly selected bulk tank milk samples from Danish dairy herds were antibody positive.

The aim of the present study was to determine the prevalence and geographical distribution of *C. burnetii *antibody positive randomly selected dairy herds in Denmark.

The survey was designed as a cross sectional study of a sample of 100 randomly selected dairy herds. As there was no prior knowledge about the herd infection frequency at the time of planning the study, the necessary sample size was based on an assumed prevalence of p = 0.50 and with an allowable error on the estimate of l = 0.10 at the 95% confidence level. Using the formula n = Z^2^pq/l^2 ^resulted in an estimated need of 96 herds. Each of the 4785 milk producing dairy herds in Denmark by 1 February 2008 was assigned a random number between 0 and 1 (SAS function Ranuni (0)) [10], and the 150 herds with the lowest numbers were chosen for the study. The only inclusion criterion was that the herd was delivering milk to a dairy plant at the time of selection.

A letter of introduction and encouragement to participate was sent to each farmer from the Danish Dairy Board. A follow up telephone call was made within 10 days to ask for the farmers' participation and cooperation starting with herds with the lowest numbers. After 117 inquiries 100 farmers had accepted.

Each farmer was mailed a letter of instruction and a 10 cc plastic tube to be filled with a representative bulk tank milk sample. The farmers were instructed to make sure that the tank milk was thoroughly stirred and cooled before sampling with a clean ladle through the hole in the top cover of the milk tank. The samples were sent to the laboratory by ordinary mail. The milk samples were collected from late February to early June 2008.

Upon arrival to the laboratory the samples were centrifuged, the fat fraction was removed and discarded, and the non-fat fraction was frozen to be tested for antibodies at a later time. The samples were tested for antibodies against *C. burnetii *using the commercial CHEKIT Q-Fever Antibody ELISA Test Kit (IDEXX, Liebefeld-Bern, Switzerland) based on *C. burnetii *inactivated phase 1 and phase 2 antigens. All samples were tested in duplicates, and the optical density (OD) of the samples were averaged and corrected by subtracting the OD of the negative control. The remaining non-fat fraction of the milk samples was frozen and stored for possible later purposes.

The results were expressed as S/P values and estimated as the ratio between OD of the sample (S) and the OD of positive control (P) included in the test kit. The S/P values were estimated on a continuous scale with a theoretical range from zero to +8. According to the instructions from IDEXX S/P ≥ 40% was considered positive, S/P < 30% was considered negative, and results in the interval 30% ≤ S/P < 40% were considered to be intermediate.

The laboratory results were entered into SAS for estimating the prevalence overall and according to geographic regions of Denmark.

**Figure 1:**
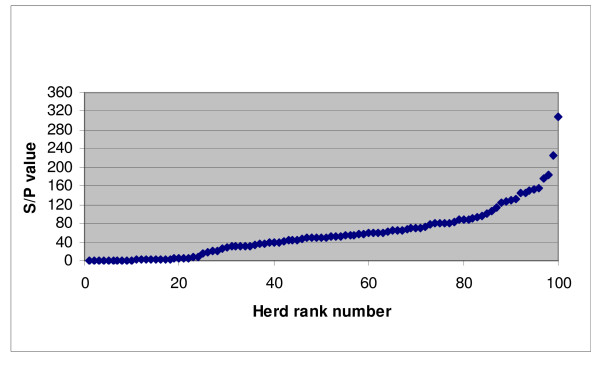
**Array of antibody S/P values to *Coxiella burnetii *in bulk tank milk samples from 100 randomly selected Danish dairy herds in the spring of 2008**.

Analysis of the bulk tank milk samples demonstrated S/P values ranging from 0 to 308 (Figure 1). The apparent prevalence of positive herds was 59%, of negative herds 30% and of intermediate herds 11% (Table 1). Further descriptive statistics for the three test categories are given in Table 1.

**Table 1:** Descriptive statistics on the presence of *Coxiella burnetii *antibodies (S/P values) measured as prevalence of test positive, test intermediate and test negative results in 100 Danish dairy herds in the spring of 2008.

Test category	No. of herds	Apparent prevalence	95% CI (p)	Range of S/P values	Mean S/P value
Positive	59	0.59	[0.49; 0.69]	42-308	88.9

Intermediate	11	0.11	[0.05; 0.17]	30-39	34.4

Negative	30	0.30	[0.21; 0.39]	0-28	6.4

**Figure 2:**
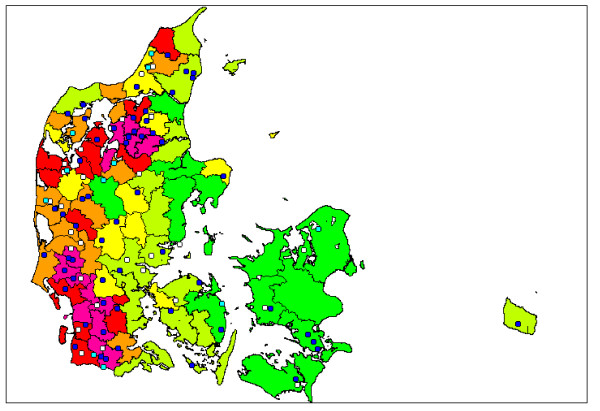
**Geographical distribution of 100 randomly selected Danish dairy herds tested for antibodies against *Coxiella burnetii *in the spring of 2008**. The herds were categorized as either test positive (S/P > 40%) (Blue colour), test intermediate (30% ≤ S/P ≥ 40%) (Turquoise colour) or test negative (S/P < 30%) (White colour). The regional dairy herd density is presented by the following colours: Green: <0.07; Pale green: 0.07-0.10; Yellow: 0.10-0.14; Orange: 0.14-0.16; Red: 0.16-0.23; Purple: >0.23 herds/km^2^.

The 100 herds were geographically representing all major areas of Denmark (Figure 2), reflecting the use of random sampling. Test positive herds were detected in all regions of Denmark, but the majority was identified in the western part of Jutland where most dairy herds are located. Figure 2 also shows the dairy herd density (herds/km^2^) in 78 geographic areas of Denmark. This leads to the hypothesis of an association between regional dairy herd density and the prevalence of *C. burnetii *antibody positive herds. The 78 areas were collapsed into 6 regions and categorized as either a low or a high density region. A preliminary test for an association between region and herd test category (59 test positive and the 30 test negative herds, thus excluding the 11 test intermediate herds) using Fisher's exact test did not indicate any significant association between regional herd density and prevalence of test positive herds. A test for association between high or low herd density and herd test category did also not indicate any association.

The study confirms that antibody positive herds are prevalent in Denmark. This was expected, as laboratory analyses of selected samples submitted for routine diagnostics by Danish veterinarians and from testing of animals intended for export during recent years have demonstrated widespread occurrence of antibody positive herds and animals in Denmark [8,9]. Both studies [[8] and [9]] were based on the CHEKIT Q-fever test kit, like in the present study.

In an attempt to characterize the spreading of C. *burnetii *infection in Danish dairy herds during the recent years, Christoffersen ([8] and additional personal communication) estimated a herd prevalence of 22.2% for year 2003 based on 164 individual samples from 90 cattle herds. For year 2004, 15 herds of 40 herds (37.5%) tested antibody positive. The samples from 2003 and 2004 were selected from herds with reproduction problems, thus not being a random sample of Danish cattle herds. For the period November 2004 to November 2006 a total of 266 blood samples were received from 133 herds with reproductive problems; of these, 66 herds had test positive animals, corresponding to 49.6% herds. For the year 2007, 57% of 753 non-randomly selected dairy herds were antibody positive on bulk tank milk [9].

As the above mentioned estimates were based on herds with a clinical suspicion of infection with C. *burnetii *or another infectious agent as e.g. *Neospora caninum*, it is reasonable to assume that the true population prevalences during the years 2003-2007 were lower than the above mentioned high risk sample estimates. When comparing to the present study result, this indicates an increasing trend in the prevalence of antibody positive herds from 2003 to 2008.

The present study included samples from herds in all regions of Denmark and was sufficiently large to estimate the prevalence. However, the sample size is too small for more detailed analysis of the geographical diversity in prevalence of positive herds. This may be the reason for non-significant relationships between regional dairy herd density and prevalence of test positive herds.

The frequency estimates are apparent prevalences. This implies that the true prevalences may be different if the test sensitivity and test specificity are less than 100%. However, the sensitivity and the specificity are unknown. Comparison of the test with the widely used complement fixation test (CF) showed good agreement in some studies, e.g. [11] and less good agreement in other studies, e.g. [12]. However, as the ELISA test is generally considered better than the CF test [13], an estimation of sensitivity and specificity of the ELISA test compared to CF as a gold standard is not relevant. More detailed studies on this are needed.

Farmers' reasons for refusing to participate were "the farmer did just not want to participate", "the farmer had no time", "the farmer was sick", and "soon closing down milk production".

Refusals to participate in the study may induce selection bias. However, we did not find any indications that the 17 farmer refusals were related to suspicions of presence of *C. burnetii *infections or any other health related issues in these farms. Selection bias of the estimates due to non-participating is therefore considered negligible.

It is concluded that there is a high prevalence of antibody positive dairy herds in Denmark. There is a relatively large number of herds with intermediate values, indicating that some of these herds may be undergoing an infection. Comparison of the results to previous Danish findings indicates that infection with *C. burnetii *may be considered to be an increasing infectious problem in Denmark. Analyses did not indicate any significant relationship between the regional herd density and the prevalence of test positive herds.

## Competing interests

The authors declare that they have no competing interests.

## Authors' contributions

JFA, ABC, ER and JSA planned the study. All authors participated in the interpretation of results and commented the manuscript. JFA selected the herds, performed the epidemiological part of the study and drafted the manuscript. ABC was responsible for sampling of milk and performed the serological examinations. JN extracted data from the Danish Cattle Database and made the cartographic presentation. All authors read and approved the final manuscript.
